# miR‐137 and its target T‐type Ca_V_3.1 channel modulate dedifferentiation and proliferation of cerebrovascular smooth muscle cells in simulated microgravity rats by regulating calcineurin/NFAT pathway

**DOI:** 10.1111/cpr.12774

**Published:** 2020-02-08

**Authors:** Bin Zhang, Li Chen, Yun‐Gang Bai, Ji‐Bo Song, Jiu‐Hua Cheng, Hong‐Zhe Ma, Jin Ma, Man‐Jiang Xie

**Affiliations:** ^1^ Department of Aerospace Physiology Key Laboratory of Aerospace Medicine of Ministry of Education Fourth Military Medical University Xi'an China

**Keywords:** dedifferentiation, microRNA, simulated microgravity, T‐type calcium channel, vascular smooth muscle cells (VSMCs)

## Abstract

**Objectives:**

Postflight orthostatic intolerance has been regarded as a major adverse effect after microgravity exposure, in which cerebrovascular adaptation plays a critical role. Our previous finding suggested that dedifferentiation of vascular smooth muscle cells (VSMCs) might be one of the key contributors to cerebrovascular adaptation under simulated microgravity. This study was aimed to confirm this concept and elucidate the underlying mechanisms.

**Materials and Methods:**

Sprague Dawley rats were subjected to 28‐day hindlimb‐unloading to simulate microgravity exposure. VSMC dedifferentiation was evaluated by ultrastructural analysis and contractile/synthetic maker detection. The role of T‐type Ca_V_3.1 channel was revealed by assessing its blocking effects. MiR‐137 was identified as the upstream of Ca_V_3.1 channel by luciferase assay and investigated by gain/loss‐of‐function approaches. Calcineurin/nuclear factor of activated T lymphocytes (NFAT) pathway, the downstream of Ca_V_3.1 channel, was investigated by detecting calcineurin activity and NFAT nuclear translocation.

**Results:**

Simulated microgravity induced the dedifferentiation and proliferation in rat cerebral VSMCs. T‐type Ca_V_3.1 channel promoted the dedifferentiation and proliferation of VSMC. MiR‐137 and calcineurin/NFATc3 pathway were the upstream and downstream signalling of T‐type Ca_V_3.1 channel in modulating the dedifferentiation and proliferation of VSMCs, respectively.

**Conclusions:**

The present work demonstrated that miR‐137 and its target T‐type Ca_V_3.1 channel modulate the dedifferentiation and proliferation of rat cerebral VSMCs under simulated microgravity by regulating calcineurin/NFATc3 pathway.

## 1 INTRODUCTION

Postflight orthostatic intolerance, associated with high risk to astronaut's health and performance, has been considered as one of the major adverse effects when exposed to microgravity environment, and there are still no effective countermeasures.^1^, ^2^ It has been reported that multiple mechanisms are implicated in the occurrence of postflight orthostatic intolerance, including hypovolaemia, alterations in baroreceptor reflex, decreases in exercise tolerance and aerobic fitness, and cardiovascular dysfunction.^3^, ^4^ Recently, both human studies from spaceflights and head‐down tilt bed tests and animal studies from tail‐suspended hindlimb‐unweighting rat model revealed that functional and structural adaptation in cerebral arteries including the augmented myogenic tone, the enhanced arterial reactivity, increased media thickness and the number of smooth muscle cell layers, could be one of the fundamental causes in the postflight orthostatic intolerance, but the underlying mechanisms remain to be clarified.^5^, ^6^

Vascular smooth muscle cell (VSMC) displays a remarkable plasticity in different phenotypes, which is crucial to maintain vascular function normally.^7^ The majority of VSMCs are stable in differentiated contractile phenotype in physiological conditions. These differentiated contractile VSMCs could undergo a rapid shift to dedifferentiated synthetic phenotype when exposed to environmental stimuli, which known as dedifferentiation or phenotype switching.^8^ During the process of dedifferentiation, VSMC lose the contractile ability, but restart the programme of cell growth, synthesis, proliferation, migration and secretion. It has been demonstrated that dedifferentiation of VSMC is characterized by reduced expression of contractile‐specific proteins, such as smooth muscle α‐actin (SM‐α‐actin), smooth muscle myosin heavy chain (SM‐MHC), smoothelin, calponin and smooth muscle protein 22α (SM22α), and increased expression of some early‐response genes, including c‐Fos, osteopontin (OPN) and proliferating cell nuclear antigen (PCNA).^7^, ^8^ Dedifferentiation of VSMC is an initial and key incident in series of cardiovascular physiologies and pathologies, such as angiogenesis, pregnancy, injury repair, atherosclerosis, hypertension and artery stenosis.^9^, ^10^ We previously observed that simulated microgravity could induce dedifferentiation and proliferation of VSMCs in the basilar arteries of rats, suggesting that it may be one of the key contributors to cerebrovascular adaptation when exposed to microgravity.^11^

The intracellular Ca^2+^, tightly controlled by Ca^2+^ channels and transporters, is an important messenger in VSMC dedifferentiation.^12^ One of the most important components of Ca^2+^ signalling in VSMC is the extracellular Ca^2+^ influx through voltage‐dependent Ca^2+^ channels (VDCCs). The high‐voltage activated L‐type (large or long‐lasting) and low‐voltage activated T‐type (tiny or transient) Ca^2+^ channels are the two main types of VDCCs in VSMC.^12^, ^13^ L‐type VDCCs has been reported to be significantly increased in differentiated contractile VSMCs and decreased in dedifferentiated synthetic VSMCs and suppressed the dedifferentiation of VSMC.^13^, ^14^ Our previous study demonstrated that simulated microgravity could upregulate the expression of L‐type VDCCs in dedifferentiated synthetic VSMCs of rat cerebral arteries,^15^ suggesting there are other Ca^2+^ signal pathways responsible for the dedifferentiation of cerebral VSMCs under simulated microgravity. Recent studies revealed that T‐type VDCCs were increased in dedifferentiated synthetic VSMCs.^8^, ^16^ We also found that the expression of T‐type VDCCs were upregulated in dedifferentiated cerebral VSMCs of simulate microgravity rats. However, the role of T‐type VDCCs in modulating the dedifferentiation of VSMC has not been elucidated up to now.

Vascular smooth muscle cells phenotype which modulated by Ca^2+^ signal is defined by Ca^2+^‐dependent transcription factors, including serum response factor (SRF), cAMP response element‐binding protein (CREBP), nuclear factor of activated T lymphocytes (NFAT) and C‐terminus of voltage‐dependent L‐type Ca^2+^ calcium channels. Different types of Ca^2+^ signalling could activate different Ca^2+^‐dependent transcription factors and thus modulate VSMC dedifferentiation by regulating the expression patterns of contractile or synthetic marker genes.^17^ Moreover, a series of specific microRNAs (miRNAs) have also been identified as the upstream signals of intracellular Ca^2+^ signalling in VSMC dedifferentiation.^8^, ^18^ Interestingly, our research showed that simulated microgravity upregulated the expression of T‐type VDCCs in a post‐transcriptional way, indicating a potential role for miRNAs in it. In this study, we aim to investigate the role of T‐type VDCCs in VSMC dedifferentiation and proliferation under simulated microgravity and its underlying mechanisms.

## 2 MATERIALS AND METHODS

### 2.1 Animal model

All animal procedures described in this study were in adherence with the Guide for the Care and Use of Laboratory Animals published by the US National Institutes of Health, with approval from Committee on the Ethics of Animal Experiments of Fourth Military Medical University. Sprague Dawley rats were subjected to a 4‐week tail suspension and hindlimbs‐unloading to simulate the cardiovascular effects of microgravity as previously described.^15^ The soleus/body weight radio was measured to assess simulated microgravity efficiency in tail‐suspended (SUS) rats routinely.

### 2.2 Isolation of cerebral VSMCs

Isolation of cerebral VSMCs was carried out as previously described.^15^ Briefly, the cerebral arteries were carefully removed and placed in 4°C physiological salt solution (PSS). For electrophysiological using, cerebral arteries were digested for 18 minutes at 37°C with solution containing 4 mg/mL papain (Biochrom), 2 mg/mL dithioerythritol (Amresco), 1 mg/mL bovine serum albumin (MP Biomedicals) and 5 mmol/L taurine in PSS. Isolated VSMCs were stored at 4°C for use within 8 hours.

### 2.3 Transmission electron microscopy

Basilar arteries were fixed, dehydrated and embedded in epoxy resin. Ultrathin sections were obtained with a diamond knife using ultramicrotome (EMUC6/FC6; Leica Microsystems) and stained with lead citrate and uranyl acetate. After that, the sections were viewed and photographically recorded using a scanning TEM (Tecnai G2 F20S‐TWIN; FEI) at 100 kV with a high‐performance Gatan CCD camera. For morphological assessments, individual cerebral VSMCs which reside in the tunica media of the basilar arterial were observed after being magnified 6000 times. Intracellular myofilaments, which support contractile apparatus, and cytoplasmic organelles including endoplasmic reticulum and mitochondria, which support synthetic apparatus, were observed after being magnified 20 000 times.

### 2.4 Immunohistochemical staining

Basilar artery segments were dissected along with partial brain, embedded and sectioned. Immunohistochemical staining was then performed following protocols ZSGB‐bio (ZSGB‐bio) recommended. Briefly, sections were incubated with appropriate dilution of the primary antibodies against SM‐MHC (Abcam), SM‐α‐actin (Abcam), SM22α (Proteintech), OPN (Abcam) and PCNA (Abcam), and the secondary IgG antibodies and horseradish peroxidase (HRP)‐conjugated streptavidin. Finally, 3,3′‐diaminobenzidine (DAB) was used as a chromogen to detect with Olympus IX71 inverted microscope. To assess nonspecific staining, sections were incubated in PBS without primary antibody as the negative control. Relative optical density (ROD) was calculated by normalizing integrated optical density (IOD) to vessel wall area. All image analyses were performed using Image Pro Plus 6.0 software.

### 2.5 Western blotting

Cerebral arteries and A7r5 cells lysis buffer were prepared in M‐PER Mammalian Protein Extraction Reagent (Thermo) with freshly 1% protease inhibitor cocktail (Thermo). After centrifugation, supernatants were denatured for Western blotting. Proteins were separated using NuPAGE 4%–12% Bis‐Tris gel (Invitrogen), and then transferred to polyvinylidene fluoride (PVDF) membranes (Millipore). Membranes were blocked and subsequently incubated with appropriate primary antibodies against SM‐MHC (Abcam), SM‐α‐actin (Abcam), SM22α (Proteintech), OPN (Abcam), PCNA (Abcam), Ca_V_3.1 (Alomone labs), Ca_V_3.2 (Affinity), NFATc1‐4 (Bioss), glyceraldehyde‐3‐phosphate dehydrogenase (GAPDH) (Proteintech) and Histone‐H3 (Abcam) at 4°C overnight and horseradish peroxidase (HRP)‐conjugated secondary antibodies (Abcam). Proteins were detected and visualized using the chemiluminescent HRP substrate (Millipore). Image J software was applied for quantification.

### 2.6 Real‐time quantitative reverse transcription polymerase chain reaction (qRT‐PCR)

Briefly, cerebral arteries and A7r5 cells was mixed with RNAiso (Takara) and homogenized by grinding. After centrifugation, phase separation and precipitation, the resulting RNA pellet was dissolved in RNase‐free water and stored at −80°C until further analysis. After reverse transcription by using Mir‐X miRNA First‐Strand Synthesis Kit (Takara), qRT‐PCR was performed using a CFX96 (BIO‐RAD) instrument and SYBR Premix Ex TaqTM (Takara) according to the manufacturer's protocol. The data were analysed via the ΔΔCt method. The PCR primers used were listed in Table1.

**Table 1:** The sequences of primers, oligonucleotides and plasmids

Name	Sequence (5′‐3′)
SM‐MHC‐forward	GATGTGGTGCAGAAAGCTCA
SM‐MHC‐reverse	CTTTGTTCACACGGCTGAGA
SM‐α‐actin‐forward	CAGGGAGTGATGGTTGGAAT
SM‐α‐actin‐reverse	GGTGATGATGCCGTGTTCTA
SM22α‐forward	GCCGTGACCAAGAACGAT
SM22α‐reverse	CTCTGTTGCCCATTTG
OPN‐forward	GTGGTTTGCTTTTGCCTGTT
OPN‐reverse	GCATCTGAGTTGCTGTAATG
PCNA‐forward	AGGACGGGGTGAAGTTTTCT
PCNA‐reverse	CAGTGGAGTGGCTTTTGTGA
Ca_V_1.2‐forward	GCGGAAGCGGCAGCAGTATG
Ca_V_1.2‐reverse	TCAGAGTCAGGCAGAGCAGAGC
Ca_V_3.1‐forward	CACCTGCTACAACACCGTCATCTC
Ca_V_3.1‐reverse	GCCTCCAACTCCGCCTCCTC
GAPDH‐forward	ATGACTCTACCCACGGCAAG
GAPDH‐reverse	TACTCAGCACCAGCATCACC
miR‐137‐forward	ACACTCCAGCTGGGTTATTGCTTAAGAA
miR‐9‐forward	ATAAAGCTAGATAACCGAAAGT
miR‐26‐forward	ACACTCCAGCTGGGTTCAAGTAATCC
miR‐96‐forward	TTTGGCACTAGCACATTTTTG
miR‐150‐forward	ATTCTCCCAACCCTTGTAC
miR‐297‐forward	ATGTATGTGTGCATGTATGCA
miR‐339‐forward	ACACTCCAGCTGGGTGAGCGCCTCGACGA
miR‐376‐forward	GGTAGATTCTCCTTCTATGA
all miRNAs‐reverse	TGGTGTCGTGGAGTCG
U6‐forward	CTCGCTTCGGCAGCACA
U6‐reverse	AACGCTTCACGAATTTGCGT
SiCa_V_3.1 sense	GCAGUUCUCCGUGUCCAAATT
SiCa_V_3.1 antisense	UUUGGUCUCGGAGAACUGCTT
miR‐137 mimic sense	UUAUUGCUUAAGAAUACGCGUAG
miR‐137 mimic antisense	CUACGCGUAUUCUUAAGCAAUAA
miR‐137 inhibitor	CUACGCGUAUUCUUAAGCAAUAA
CACNA1G 3′UTR WT	CTCGAGCCCGACACCAGGAGCTGTTGGGAGAAAGCAATACGTTTGTGCAGAATCTCTATGTATATTGCGGCCGC
CACNA1G 3′UTR MUT	CTCGAGCCCGACACCAGGAGCTGTTGGGAGAATAGACACCGTTTGTGCAGAATCTCTATGTATATTGCGGCCGC

### 2.7 Electrophysiological recordings

Currents were recorded using the whole‐cell patch‐clamp technique in isolated cerebral arteries VSMCs as described previously.^19^ Command‐voltage protocols and data acquisition were performed with pCLAMP software (version 8.0, Axon Instruments). To account for differences in cell size, currents were normalized to Cm to obtain the current densities. Currents were filtered at 0.5 kHz and digitized at 4 kHz. Ba^2+^ replaced Ca^2+^ as charge carrier to increase unitary currents and to minimize Ca^2+^‐dependent run‐down. To obtain the I–V curve of T‐type VDCCs, the current densities were plotted against the corresponding command potentials. Two kinds of external solutions, solutions A and B, were used. Solution A was used while making a gigaohm seal between the recording pipette and cell surface. It contained (in mmol/L) 130 NaCl, 5.4 KCl, 1 MgCl_2_, 10 BaCl_2_, 10 HEPES and 10 glucose, equilibrated with 95% O_2_‐5% CO_2_ at pH 7.4 titrated with NaOH. After a seal of 2 GΩ was obtained, the perfusion fluid was changed to solution B during current recording. Solution B contained (in mmol/L) 75 Tris‐Cl, 50 BaCl_2_, 10 HEPES and 10 glucose, equilibrated with 95% O_2_‐5% CO_2_ at pH 7.4 titrated with Tris base. Cs^+^ was used in the pipette solution to minimize outward K^+^ current. The pipette contained (in mmol/L) 150 CsCl, 1 MgCl_2_, 10 EGTA, 5 HEPES, 5 Na_2_ATP and 5 Na_2_ creatine phosphate, equilibrated with 95% O_2_‐5% CO_2_ at pH 7.2 titrated with CsOH. Nifedipine and mibefradil have been reported to block L‐type and T‐type VDCCs with an IC50 of 0.01 µmol/L and 0.12‐0.14 µmol/L.^20^ Application of 0.1 μmol/L nifedipine and 5 μmol/L mibefradil was used in this study to identify the properties of L‐ and T‐type VDCC channels.

### 2.8 Calcineurin phosphatase activity assay

The activity of calcineurin was determined using a calcineurin activity assay kit following the manufacturer's protocol (Genmed). Calcineurin activity was assessed by measuring the absorbance value at 660 nm using the following formula: calcineurin activity (U/mg prot) = [(testing tube OD value − control tube OD value)/standard tube OD value – standard blank tube OD value] × concentration of standard tube × dilution multiple of reaction system/sample protein concentration, the value in control group was set to one.

### 2.9 Cell culture

A7r5 cells (rat thoracic aortic smooth muscle cell line) were purchased from the Chinese Academy of Sciences (Shanghai, China) and cultured in DMEM (Hyclone) supplemented with 10% foetal bovine serum (FBS) (Gibco), 100 U/mL penicillin (Solarbio), and 100 µg/mL streptomycin (Solarbio). To induce dedifferentiation of VSMCs in vitro, serum stimulation was given to A7r5 cells after 48 hours serum starvation (cultured in serum‐free medium) by incubated with 10% FBS, as previously described.^21^

### 2.10 Oligonucleotides transient transfection

Oligonucleotides including siRNA (target CACNA1G sequence, SiCa_V_3.1), siRNA control (SiNC), miR‐137 mimic/inhibitor and corresponding controls (miR‐NC) were all purchased from Ribobio Corporation. The sequences were listed in Table. 1. According to the manufacturer's instructions, A7r5 cells were transfected with SiCa_V_3.1 (100 nmol/L) or miR‐137 mimic/inhibitor (50 nmol/L/150 nmol/L), and their control using Lipofectamine 3000 reagent (Invitrogen) and harvested after 48 hours transfection.

### 2.11 Dual‐luciferase report assay

PsiCHECKTM‐2 vector (Promega) containing both Firefly and Renilla luciferase genes was used to introduce the wild/mutant 3′UTR sequences of Cacna1g immediately downstream the stop codon of the Renilla luciferase gene to create a wild‐type (WT) or mutant‐type (MUT) CACNA1G 3′UTR plasmid (Sangon Biotech). PsiCHECKTM‐2 vector without inserted gene was used as negative control (NC) plasmid. The inserted sequences were listed in Table. 1. Following the procedures provided by Ribobio and Promega, A7r5 cells were co‐transfected with PsiCHECKTM‐2 vector (WT/MUT/NC) and oligonucleotides (miR‐137 mimic or mimic control) for 48 hours. The Firefly and Renilla luciferase activities (Fluc, Rluc) were sequentially measured using the Dual‐Luciferase Reporter Assay system (Promega) as recommended. Relative luciferase activity was calculated by normalizing Rluc to Fluc, the value in NC plasmid plus mimic control treated group was set to one.

### 2.12 EdU incorporation assay

Proliferation of A7r5 cells was determined by a 5‐ethynyl‐2‐deoxyuridine (EdU) incorporation assay. EdU (RiboBio) was added at 100 μmol/L, and the cells were cultured for an additional 2 hours. After the removal of EdU‐containing medium, the cells were fixed, washed with glycine, treated with TrionX‐100, reaction buffer, stained with Hoechst and examined under a fluorescence microscope, according to the manufacturer's instructions. The proliferation rate was calculated by normalizing the number of EdU positive cells in five random microscopic fields.

### 2.13 Statistical analysis

Data are expressed as means ± SEM. All experiments were performed at least in triplicates. Statistical analysis using Student's *t* test (two group comparison) or one‐way ANOVA (multiple group comparison) was done with Graphpad Prism software (version 5.01). A *P* value <.05 was considered to be statistically significant.

### 2.14 Chemicals and reagents

All buffers, chemicals and reagents were purchased from Sigma‐Aldrich unless otherwise stated.

## 3 RESULTS

### 3.1 General data

As shown in Table. 2, there was no significant difference in the final body weights between CON and SUS rats, indicating a normal growth rate during simulated microgravity. The ratio of soleus/body weight significantly decreased in SUS rats as compared with that in CON rats, which suggested the deconditioning effects of simulated microgravity.

**Table 2:** Body weight, soleus wet weight and the ratio of soleus/body weight in CON and SUS rats

	Body weight (g)		left soleus weight (mg)	Soleus/body weight (mg/g)
	Initial	Final		
CON (n = 50)	229.47 ± 3.52	410.83 ± 6.17	161.17 ± 2.79	0.39 ± 0.01
SUS (n = 50)	228.73 ± 4.12	402.57 ± 5.23	70.83 ± 1.60*	0.18 ± 0.01*

Abbreviations: CON, 28‐d control rats; SUS, 28‐d tail‐suspended rats.

Values of body weights are means ± SD; others are means ± SEM.

* *P* < .001 vs CON (analysed by Student's *t* test).

### 3.2 Simulated microgravity induced a dedifferentiation from contractile to synthetic phenotype in rat cerebral VSMCs

**Figure 1:**
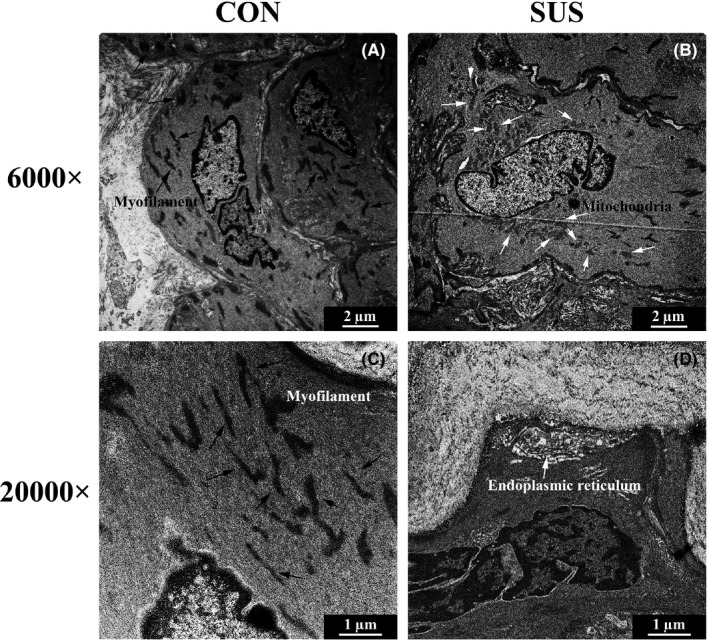
Comparisons of the ultrastructure in cerebral VSMCs of CON and SUS rats. A, C, Typical differentiated contractile VSMCs from CON rats showed numerous myofilaments (black arrows) (original magnification 6000× at A and 20 000× at C). B, D, Dedifferentiated synthetic VSMCs from SUS rats exhibited rare myofilaments and rich in cytoplasmic organelles (white arrows) including endoplasmic reticulum, mitochondria (original magnification 6000× at B and 20 000× at D)

**Figure 2:**
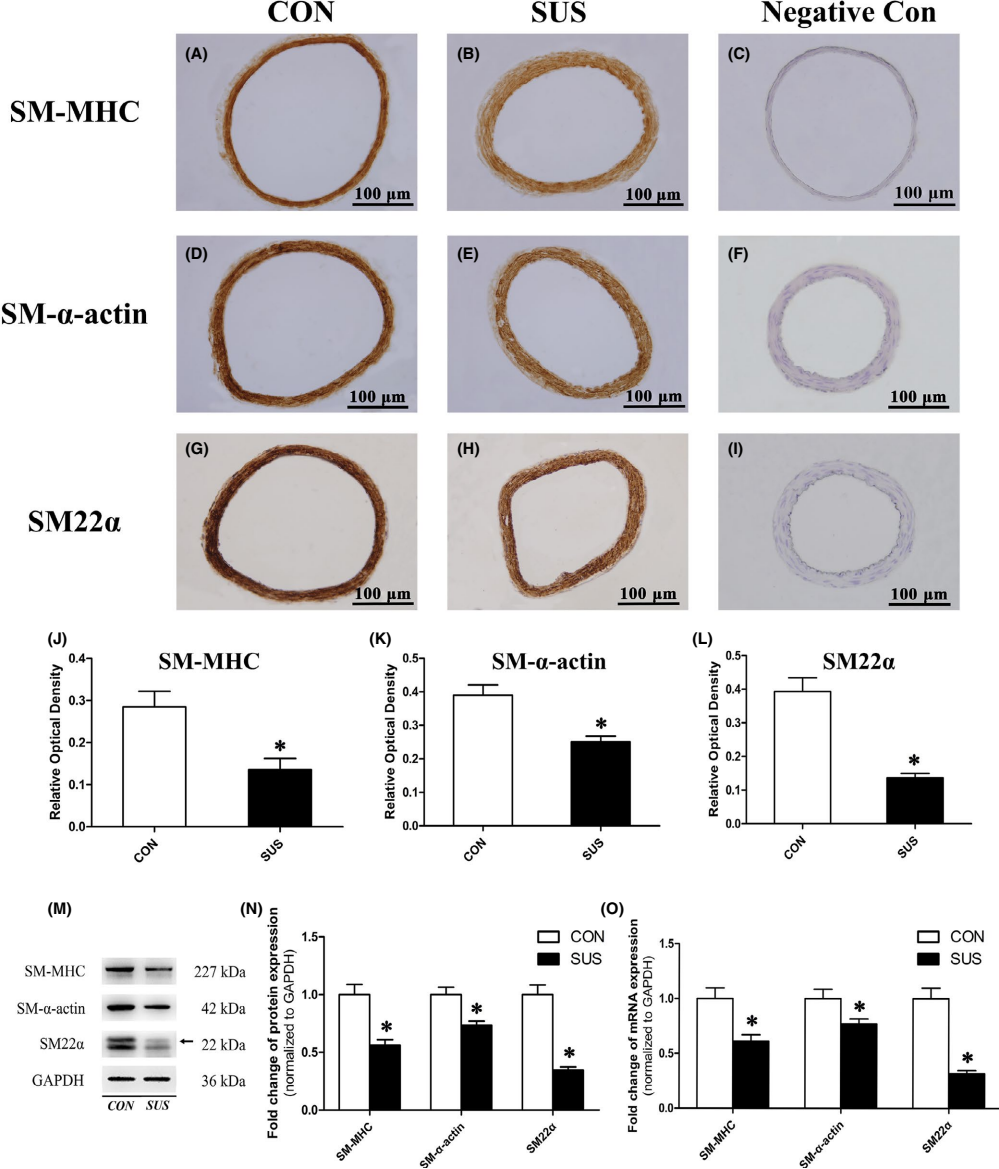
Comparisons of contractile maker expressions at protein and mRNA levels in cerebral arteries of CON and SUS rats. A‐L, Immunohistochemical staining for SM‐MHC, SM‐α‐actin and SM22α, and quantitative analysis by the relative optical density, which was calculated by normalizing integrated optical density to vessel wall area (n = 6/group). M‐N, The protein expressions of SM‐MHC, SM‐α‐actin and SM22α were examined by Western blotting. GAPDH was used as loading control. Representative Western blots (M) and densitometry analysis (N) were shown (n = 10/group). O, mRNA levels of SM‐MHC, SM‐α‐actin and SM22α were assessed by qRT‐PCR analysis. GAPDH was used for normalization (n = 10/group). Data were presented as means ± SEM. *P < .05 as compared with control (analysed by Student's *t* test)

**Figure 3:**
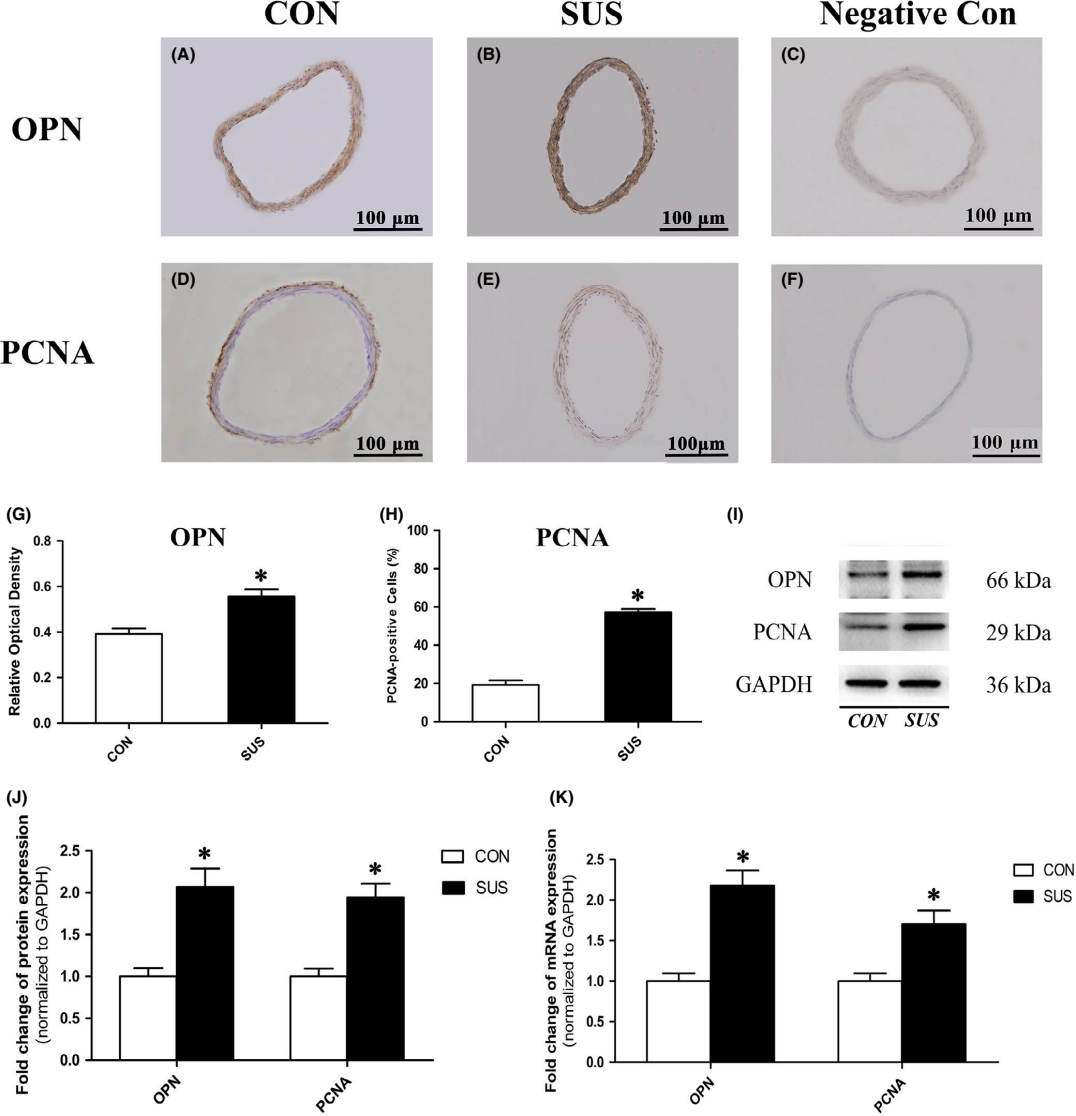
Comparisons of synthetic maker expressions in cerebral arteries of CON and SUS rats. A‐H, Immunohistochemical staining for OPN and PCNA and quantitative analysis by the relative optical density, which was calculated by normalizing integrated optical density to vessel wall area (n = 6/group). I‐J, The protein expression of OPN and PCNA was examined by Western blotting. GAPDH was used as loading control. Representative Western blots (I) and densitometry analysis (J) were shown (n = 10/group). K, mRNA levels of OPN and PCNA were assessed by qRT‐PCR analysis. GAPDH was used for normalization (n = 10/group). Data were presented as means ± SEM. *P < .05 as compared with control (analysed by Student's *t* test)

As shown in Figure 1, cerebral VSMCs in CON rats showed a typical differentiated contractile phenotype feature with abundant myofilaments (black arrows), while cerebral VSMCs in SUS rats showed a dedifferentiated synthetic phenotype feature with less myofilaments and more synthetic organelles (white arrows) including endoplasmic reticulum and mitochondria in cytoplasm. As immunohistochemical staining, Western blotting and qRT‐PCR analysis showed, the relative protein (Figure 2A‐N) and mRNA (Figure 2O) expressions of contractile markers (including SM‐MHC, SM‐α‐actin and SM22α) significantly reduced, whereas the relative protein (Figure 3A‐J) and mRNA (Figure 3K) expressions of synthetic markers (including OPN and PCNA) markedly increased in cerebral arteries of SUS rat as compared with that of CON rats. These results indicated that simulated microgravity‐induced dedifferentiation of rat cerebral VSMCs from contractile phenotype to synthetic phenotype.

### 3.3 Simulated microgravity activated T‐type Ca_V_3.1 channel in rat cerebral VSMCs

**Figure 4:**
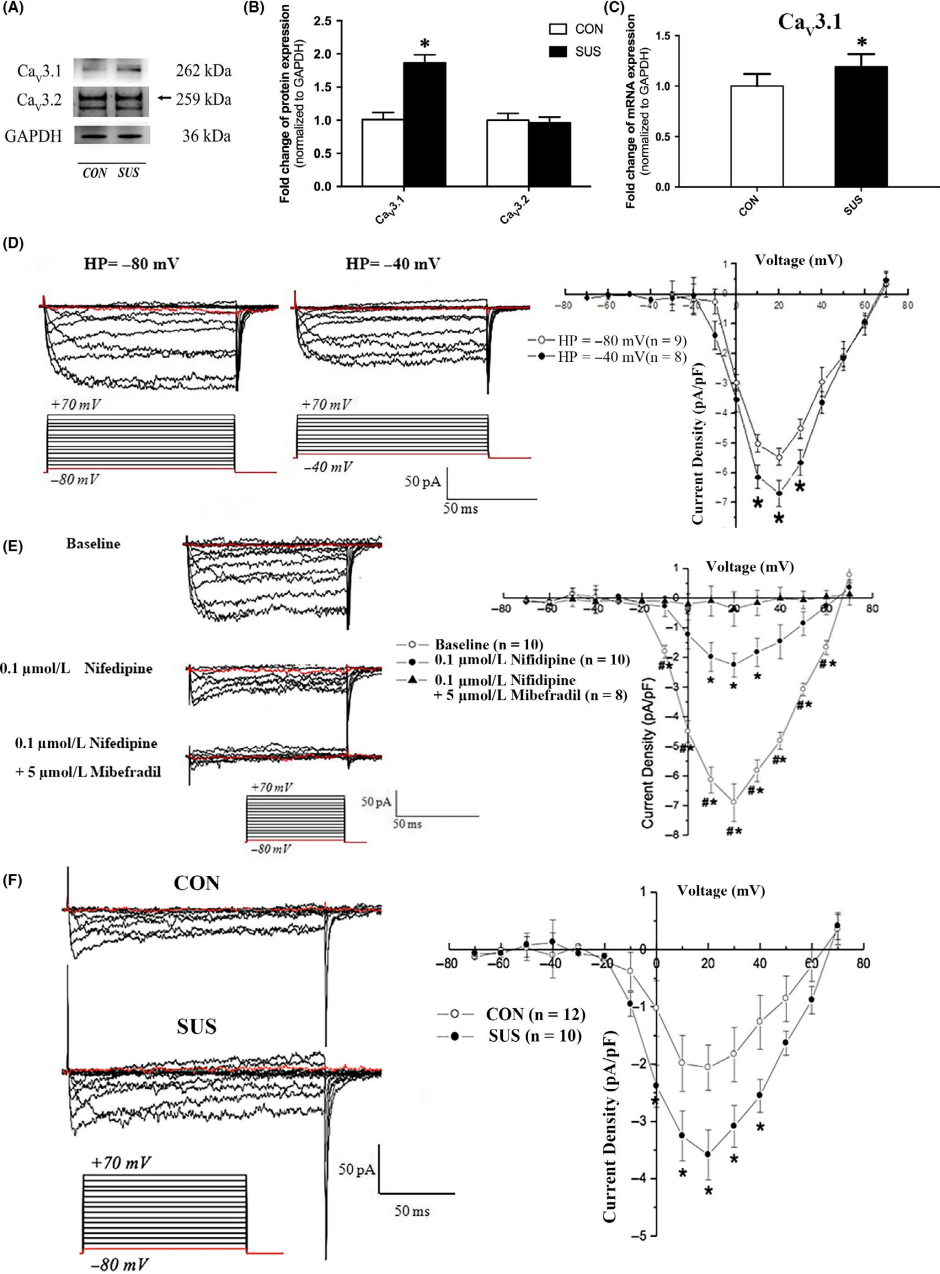
Comparisons of protein expression and whole‐cell current densities of T‐type VDCCs in cerebral VSMCs isolated from CON and SUS rats. A‐B, The protein expressions of T‐type Ca_V_3.1 and Ca_V_3.2 channels were examined by Western blotting. GAPDH was used as loading control (n = 10/group). C, mRNA levels of T‐type Ca_V_3.1 channels were assessed by qRT‐PCR analysis. GAPDH was used for normalization (n = 10/group). D, Inward whole‐cell currents were recorded from isolated cerebral VSMCs at −80 mV and −40 mV holding potentials, respectively. E, Whole‐cell currents recorded from cerebral VSMCs with the extracellular application of 0.1 μmol/L Nifedipine and 5 μmol/L Mibefradil in the presence of 0.1 μmol/L Nifedipine. Cumulative *I‐V* curves are shown in the right panel. F, Whole‐cell currents recorded at −80 mV holding potential in the presence of 0.1 μmol/L Nifedipine were used as the representative T‐type Ca_V_3.1 channels currents. The values are expressed as means ± SEM with the number of cells recorded in parentheses. Data were presented as mean ± SEM. *P < .05 vs control or Baseline, ^#^ *P* < .05 vs 0.1 μmol/L Nifedipine (analysed by Student's *t* test in B, C, D and F; analysed by ANOVA in E)

As shown in Figure 4A‐B, the protein expressions of T‐type Ca_V_3.2 channel in cerebral arteries did not show significant change between CON and SUS rats. However, the protein (Figure 4A‐B) and mRNA (Figure 4C) expressions of T‐type Ca_V_3.1 channel in cerebral arteries of SUS rat significantly increased compared with that of CON rats. As shown in Figure 4D, there was a significant difference between the HP =−80 mV and HP =−40 mV recordings, which indicated that T‐type VDCCs currents could be recorded at a more hyperpolarized holding potential, consistent with other reports.^22^ In the present work, we found that 0.1 μmol/L Nifedipine could block about 70% inward Ca^2+^ currents in rat cerebral VSMCs at HP =−80 mV as indicated in Figure 4E. Furthermore, addition of 5 μmol/L Mibefradil could block about 85% inward Nifedipine‐insensitive Ca^2+^ currents in rat cerebral VSMCs at HP =−80 mV in the presence of 0.1 μmol/L Nifedipine. Therefore, the whole‐cell currents recorded at −80 mV HP in the presence of 0.1 μmol/L Nifedipine were used as the representative T‐type VDCCs currents in our experimental system (Figure 4F). As compared with that of CON rats, the current densities of T‐type VDCCs in cerebral VSMCs of SUS rats were significantly increased. These results indicated that simulated microgravity increased the expression and function of T‐type Ca_V_3.1 channel in cerebral VSMCs.

### 3.4 T‐type Ca_V_3.1 channel promoted VSMC dedifferentiation and proliferation

**Figure 5:**
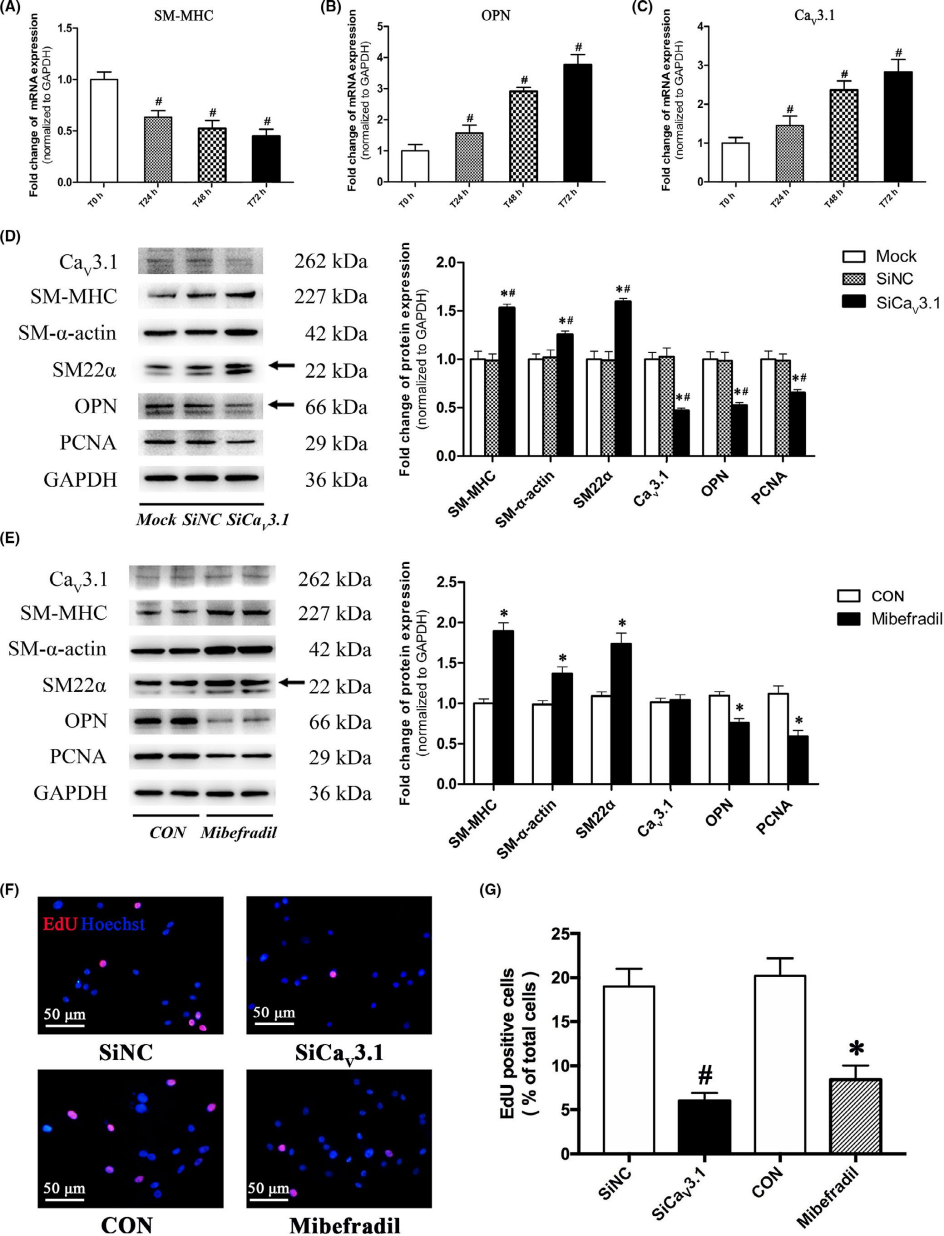
T‐type Ca_V_3.1 channel promoted VSMC dedifferentiation and proliferation in A7r5 cells. A‐C, mRNA expression of SM‐MHC (A), OPN (B), and T‐type Ca_V_3.1 channel (C) were assessed by qRT‐PCR analysis during serum stimulation induced VSMC dedifferentiation in vitro. GAPDH was used for normalization. Data were presented as mean ± SEM (n = 3/group). ^#^ *P* < .05 vs T0h (0 h after serum stimulation) (analysed by Student's *t* test). D‐E, Expression of contractile (SM‐MHC, SM‐α‐actin and SM22α) and synthetic marker (OPN and PCNA) were assessed by Western blotting under 48 h siRNA oligonucleotide transfection (D) or 48 h 5 μmol/L mibefradil treatment (E) in A7r5 cells. GAPDH was used as loading control. Data were presented as mean ± SEM (n = 3/group). *P < .05 vs control or Mock, ^#^ *P* < .05 vs SiNC (siRNA control) (analysed by ANOVA in D, Analysed by Student's *t* test in E). F‐G, A7r5 cell proliferation was assessed by an EdU incorporation assay after 48 h siRNA transfection or mibefradil treatment. Representative staining images (F) and analysis (G) were shown. Data were presented as mean ± SEM (n = 3/group). ^#^ *P* < .05 vs SiNC, *P < .05 vs control (analysed by Student's *t* test)

During the process of serum‐induced dedifferentiation in cultured A7r5 cells, the mRNA expression of contractile marker (SM‐MHC) significantly decreased, whereas synthetic marker (OPN) markedly increased in a time‐dependent manner (Figure 5A, B). Accordingly, the mRNA expression of T‐type Ca_V_3.1 was also continuously increased during this process (Figure 5C). Interestingly, whether silencing T‐type Ca_V_3.1 channel by specific siRNA oligonucleotide (Figure 5D, F, G) or blocking it by 5 μmol/L mibefradil (Figure 5E, F, G) significantly increased expressions of contractile makers (SM‐MHC, SM‐α‐actin, and SM22α), decreased expressions of synthetic makers (OPN and PCNA) and suppressed cell proliferation. These findings indicated that T‐type Ca_V_3.1 channel promoted VSMC dedifferentiation from contractile to synthetic phenotype and cell proliferation.

### 3.5 Calcineurin/NFATc3 was the downstream signalling of T‐type Ca_V_3.1 channel in VSMC dedifferentiation and proliferation

**Figure 6:**
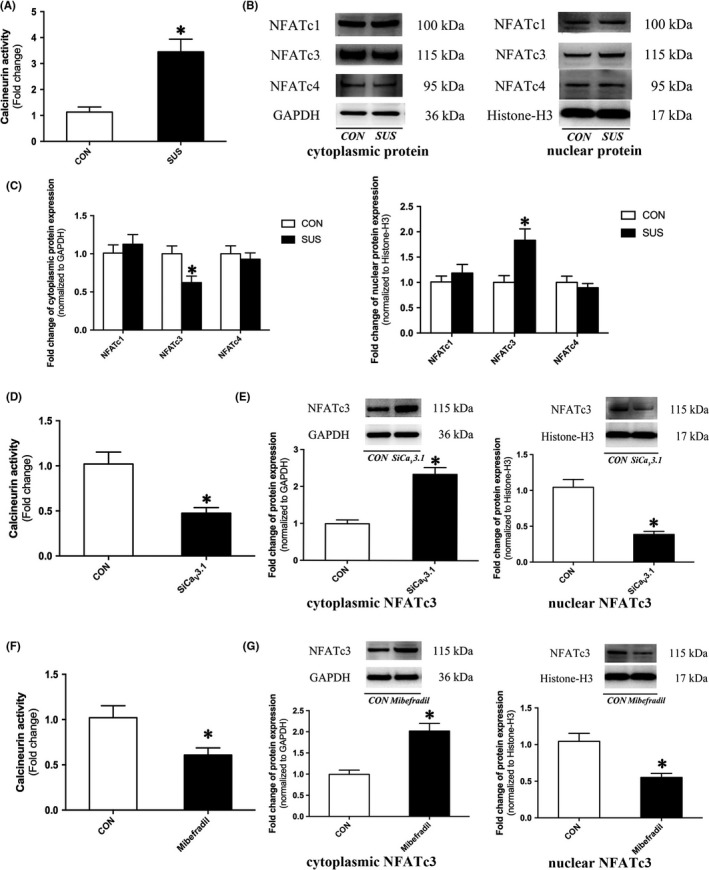
T‐type Ca_V_3.1 channel promoted VSMC dedifferentiation by activating calcineurin‐NFATc3 signalling pathway. A, Calcineurin phosphatase activity in cerebral arteries of CON and SUS rats (n = 6/group). B‐C, Expression of cytoplasmic/nuclear NFATc1‐c4 in cerebral arteries of CON and SUS rats were assessed by Western blotting. GAPDH was used as cytoplasmic protein loading control. Histone‐H3 was used as nuclear protein loading control. Representative Western blots (B) and densitometry analysis (C) were shown (n = 6/group). D, Calcineurin phosphatase activity in A7r5 cells after 48 h siCa_V_3.1 transfection (n = 3/group). E, Expression of cytoplasmic and nuclear NFATc3 in 48 h siCa_V_3.1 transfected A7r5 cells were assessed by Western blotting. GAPDH/Histone‐H3 was used as loading control (n = 3/group). F, Calcineurin phosphatase activity in A7r5 cells after 48 h mibefradil treatment (n = 3/group). G, Expression of cytoplasmic and nuclear NFATc3 in 48 h mibefradil treated A7r5 cells were assessed by Western blotting. GAPDH/Histone‐H3 was used as loading control (n = 3/group). Data were presented as mean ± SEM. *P < .05 vs control (analysed by Student's *t* test)

To investigate the downstream signalling of T‐type Ca_V_3.1 channel, Ca^2+^‐dependent calcineurin/NFAT was examined in cerebral arteries of rats. As shown in Figure 6A, calcineurin phosphatase activity was significantly increased in cerebral arteries of SUS rats as compared with that in CON rats. Considering nuclear translocation is required to activate NFAT‐dependent transcription, cytoplasmic and nuclear NFATc1‐c4 protein were extracted and quantified. Results showed that there was no significant difference about both cytoplasmic and nuclear protein expression of NFATc1 and NFATc4 in cerebral arteries between SUS rats and CON rats (Figure 6B, C). NFATc2 protein expression in cerebral arteries of SUS rats and CON rats was too low to be detected. Interestingly, NFATc3 in cerebral arteries of SUS rats markedly decreased in cytoplasmic fractions, whereas significantly increased in nuclear fractions as compared with that in CON rats (Figure 6B, C), which indicated the enhanced nuclear translocation of NFATc3 in cerebral arteries of SUS rats. In addition, silencing T‐type Ca_V_3.1 channel by specific siRNA oligonucleotide (Figure 6D, E) or blocking it by 5 μmol/L mibefradil (Figure 6F, G) significantly inhibited calcineurin phosphatase activity (Figure 6D, F) and the nuclear translocation of NFATc3 (Figure 6E, G) in serum‐induced dedifferentiated proliferative A7r5 cells. These findings suggested that calcineurin/NFATc3 signalling was T‐type Ca_V_3.1 channel dependent in VSMC dedifferentiation and proliferation.

### 3.6 miR‐137 was a negative regulator in VSMC dedifferentiation and proliferation

**Figure 7:**
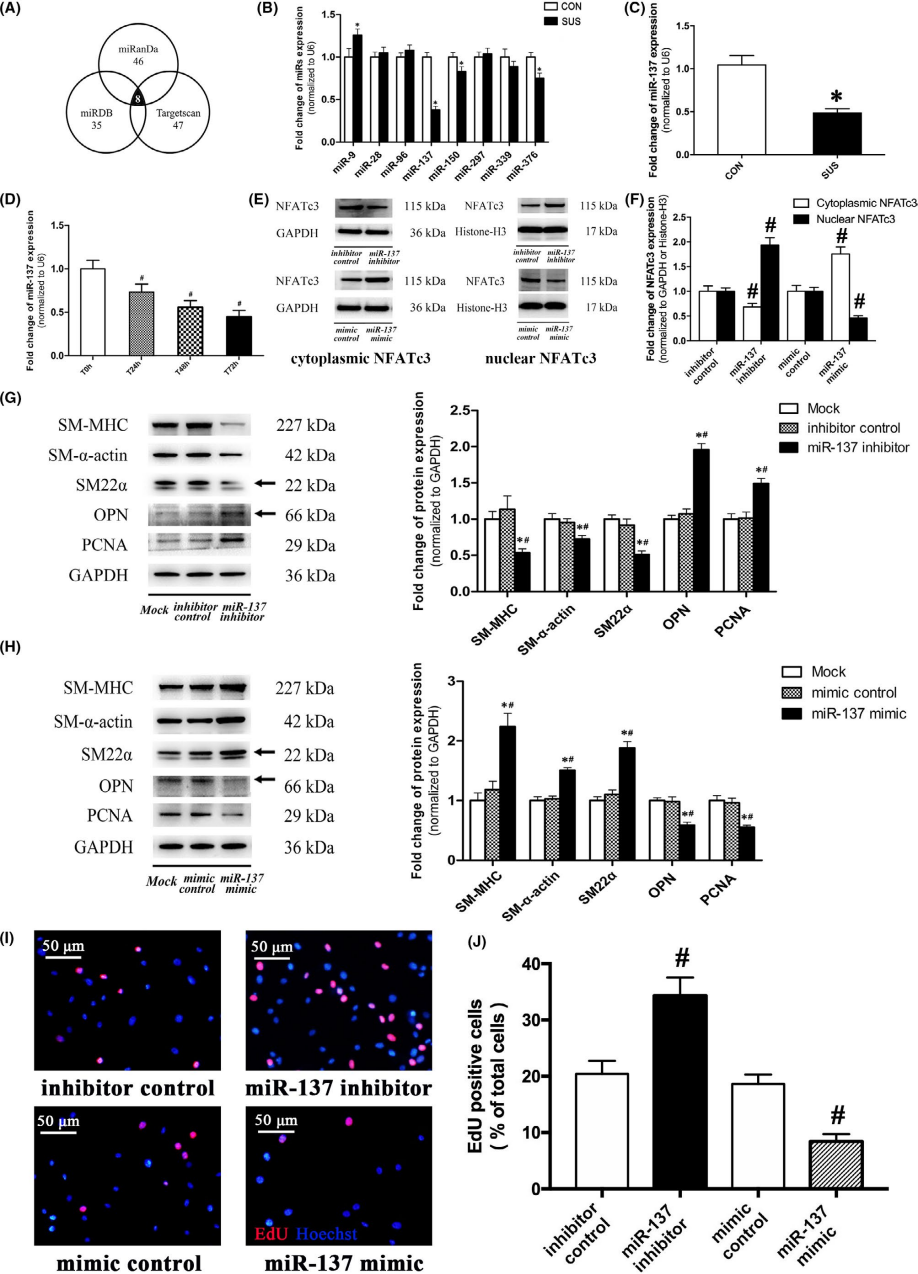
Identification of miR‐137 as a regulator in VSMC differentiation. A, Computational prediction of miRNAs, which inhibited Ca_V_3.1. B, qRT‐PCR was performed to examine the mRNA levels of eightt candidate miRNAs in cerebral VSMCs of SUS rat. Normalization was to level of U6 (n = 3/group). C, qRT‐PCR was performed to examine mRNA levels of miR‐137 in VSMCs of SUS rats in vivo. Normalization was to level of U6 (n = 10/group). D, qRT‐PCR was performed to examine mRNA levels of miR‐137 in serum stimulation induced dedifferentiated A7r5 cells in vitro. Normalization was to level of U6 (n = 3/group). E‐F, Expression of cytoplasmic and nuclear NFATc3 in 48 h miR‐137 mimic/inhibitor transfected A7r5 cells were assessed by Western blotting. GAPDH/Histone‐H3 was used as loading control. Representative Western blots (E) and densitometry analysis (F) were shown (n = 3/group). G‐H, Expression of contractile (SM‐MHC, SM‐α‐actin and SM22α) and synthetic marker (OPN and PCNA) were assessed by Western blotting after 48 h miR‐137 mimic (G) or inhibitor (H) transfection in A7r5 cells, respectively. GAPDH was used as loading control (n = 3/group). I‐J, A7r5 cell proliferation was assessed by an EdU incorporation assay after 48 h miR‐137 mimic/inhibitor transfection (n = 3/group). Representative staining images (I) and analysis (J) were shown. Data were presented as mean ± SEM. *P < .05 vs control or mock, ^#^ *P* < .05 vs T0h control (0 h after serum stimulation) or mimic/inhibitor control (analysed by Student's *t* test in B, C, F and J; analysed by ANOVA in D, G and H)

To investigate the upstream signalling of T‐type Ca_V_3.1 channel, we have screened eight candidate miRNAs related to Ca^2+^ signalling through the prediction of microRNA target software programs, including miRanda (http://www.microrna.org), miRDB (http://www.mirdb.org) and Targetscan (http://www.targetscan.org) (Figure 7A). By RT‐PCR analysis, miR‐137 was found to be the most significantly decreased miRNA among candidate miRNAs in cerebral arteries of SUS rats as compared with that in CON rats (Figure 7B). More samples were used to further confirm that miR‐137 was significantly downregulated in dedifferentiated cerebral VSMCs of SUS rats in vivo (Figure 7C) and in serum‐induced dedifferentiated A7r5 cells in vitro (Figure 7D). As shown in Figure 7E and F, miR‐137 inhibitor activated the nuclear translocation of NFATc3, while miR‐137 mimic inhibited the nuclear translocation of NFATc3, significantly. Moreover, miR‐137 inhibitor markedly decreased the protein expressions of contractile markers (SM‐MHC, SM‐α‐actin, and SM22α), whereas significantly increased the synthetic markers (OPN and PCNA) expressions (Figure 7G) and cell proliferation (Figure 7I, J) in cultured A7r5 cells. In contrast, miR‐137 mimic significantly increased the protein expressions of contractile markers, whereas markedly suppressed the synthetic markers expression (Figure 7H) and cell proliferation (Figure 7I, J) in cultured A7r5 cells. These findings suggested miR‐137 as a negative regulator in VSMC dedifferentiation and proliferation.

### 3.7 miR‐137 modulated VSMC dedifferentiation and proliferation by targeting T‐type Ca_V_3.1 channel

**Figure 8:**
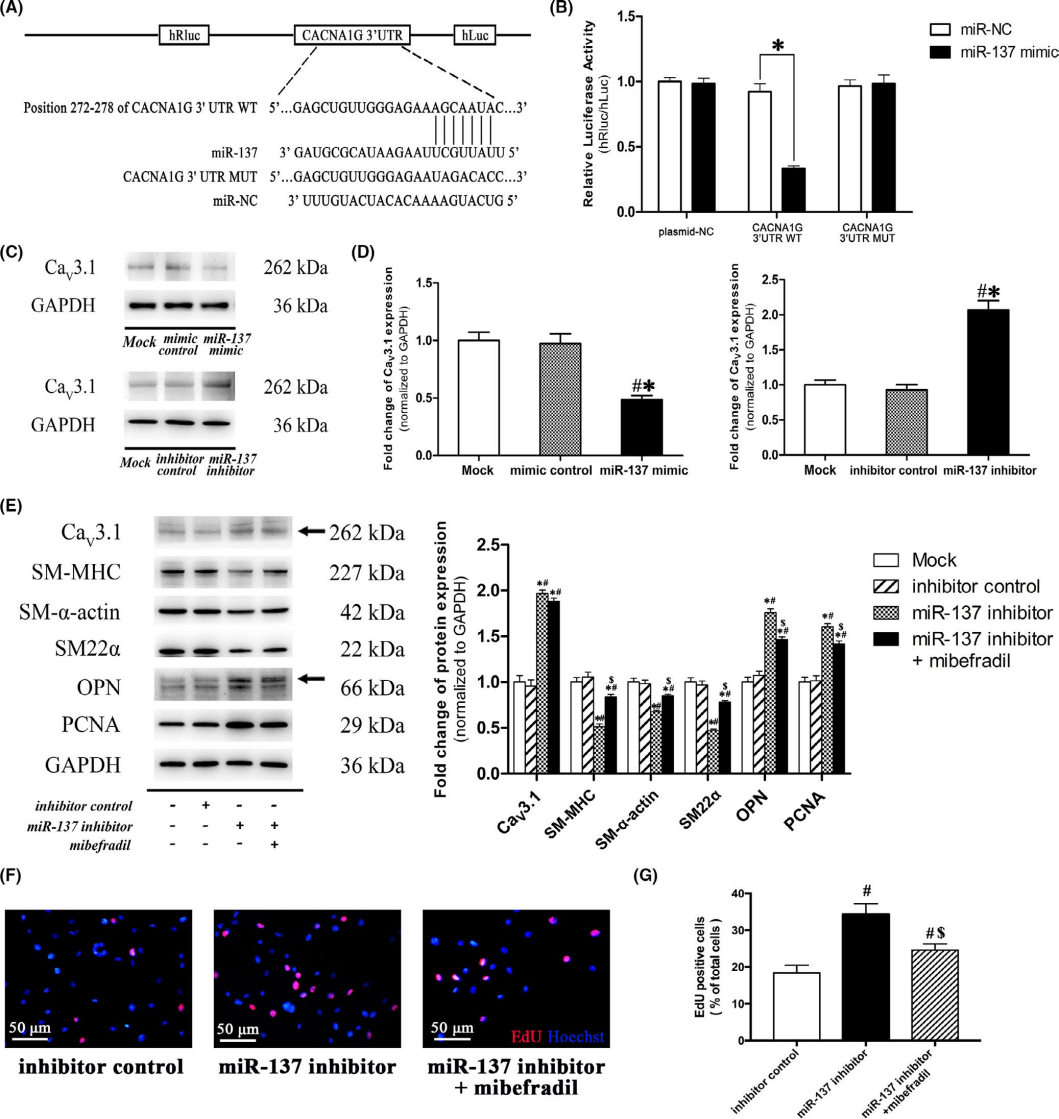
miR‐137 suppressed VSMC dedifferentiation and proliferation by targeting T‐type Ca_V_3.1 channel. A, Schematic diagram of the presumptive binding sequences of miR‐137 to *CACNA1G* 3'UTR based on the TargetScan database prediction. B, Dual‐luciferase activity assay was performed in A7r5 cells by co‐transfecting miR‐137 mimic with psiCHECK^TM^‐2 vectors containing WT/MUT *CACNA1G* 3’UTR sequences. The ratio of hRluc/hLuc were applied as relative luciferase activity. C‐D, Western blotting was performed to evaluate protein expression of T‐type Ca_V_3.1 under miR‐137 mimic or inhibitor transfection in A7r5 cells, respectively. E, Phenotype marker expressions were examined under 5 μmol/L mibefradil (the specific Ca_V_3.1 blocker) treatment in miR‐137 inhibitor induced dedifferentiated A7r5 cells. F‐G, Cell proliferation was assessed by an EdU incorporation assay under 5 μmol/L mibefradil (the specific Ca_V_3.1 blocker) treatment in miR‐137 inhibitor induced proliferative A7r5 cells. Representative staining images (F) and analysis (G) were shown. Data were presented as mean ± SEM (n = 3/group). *P < .05 vs mock, ^#^ *P* < .05 vs mimic/inhibitor control, ^$^ *P* < .05 vs miR‐137 inhibitor (analysed by ANOVA)

Dual‐luciferase reporter assay system was carried out by co‐transfecting the plasmid (WT/MUT/NC) with miR‐137 mimic or mimic control into A7r5 cells, respectively (Figure 8A, B). Results showed that miR‐137 mimic only suppressed the relative luciferase activity of CACNA1G 3′UTR WT reporter, significantly. Furthermore, the gain‐ and loss‐of‐function studies in A7r5 cells clearly showed that miR‐137 mimic significantly decreased the protein expression of T‐type Ca_V_3.1, whereas miR‐137 inhibitor significantly increased the protein expression of T‐type Ca_V_3.1 (Figure 8C, D). These results clearly demonstrated that miR‐137 negatively regulates T‐type Ca_V_3.1 channel by directly binding to CACNA1G 3′UTR complimentary sequences in VSMCs.

We found that downregulation of miR‐137 is a characteristic in VSMC dedifferentiation and proliferation in vivo and in vitro (Figure 7 C, D). Therefore, miR‐137 inhibitor was used to assess the function of miR‐137/Ca_V_3.1 in VSMC dedifferentiation and proliferation. As shown in Figure 8E, F and G, downregulation of miR‐137 by inhibitor transfection significantly increased the T‐type Ca_V_3.1 channel protein expression, accompanied with the decreased contractile markers expression and the increased synthetic markers expression and cell proliferation. Interestingly, the functional blockage of T‐type Ca_V_3.1 channel by 5 μmol/L mibefradil partially reversed the dedifferentiation and proliferation effects induced by miR‐137 inhibitor (Figure 8E, F, G). These findings clearly suggested that miR‐137 modulated VSMC dedifferentiation and proliferation by targeting T‐type Ca_V_3.1 channel.

## 4 DISCUSSION

Gravity or microgravity has a profound effect on the mechanical distribution of fluid within the cardiovascular system.^3^ There is a hydrostatic pressure gradient from the head to feet in human upright posture due to 1 G gravity. When exposed to microgravity, the hydrostatic gradients are lost throughout the vasculature, which induced a cephalad shift in fluid distribution from the lower part of the body towards the upper body.^6^ It is considered that arterial pressures around the heart level maintain relatively unchanged, but the peripheral vasculature exhibited an increased transmural pressure in the upper body and a decreased transmural pressure in the lower body during the microgravity.^3^, ^6^ The cerebral arteries would undergo adjustments due to adaptation to cerebral hypertension, which protects the down‐stream microcirculation in the brain during spaceflight. However, it also contribute to postflight orthostatic intolerance.^11^ It has been reported that a 28‐day simulated microgravity could induce a functional adaptation including enhanced myogenic tone and vasoconstrictor reactivity in middle cerebral arteries of rats.^6^, ^11^ In addition, there was a structural adaptation including thicker wall, narrower lumen and a migration of proliferated VSMCs towards the subendothelial layer in the basilar arteries of simulated microgravity rats.^6^, ^11^ Studies demonstrated that elevated transmural pressures or mechanical stretches could act directly on VSMCs and are regarded as one of the crucial factors to activate the VSMC dedifferentiation and proliferation.^8^, ^23^ For example, spontaneous hypertension induced a partial dedifferentiation from contractile to synthetic phenotype in aortic VSMCs of rats.^24^ We wonder whether the elevated transmural pressure of cerebral arteries under simulated microgravity could act on cerebral VSMCs and thus induce the dedifferentiation and proliferation of these cells, as a key initial step of microgravity‐induced structural adaptation of cerebral arteries. As indicated by the ultrastructural characteristics (Figure 1), the decreased expression of contractile markers (Figure 2) and the increased expression of synthetic markers (Figure 3) in vivo, our work clearly confirmed that simulated microgravity could induce dedifferentiation and proliferation of cerebral VSMCs, which supplemented and improved the underlying mechanisms in the development of cerebrovascular adaptation under microgravity environment exposure.

The mechanisms underlying VSMC dedifferentiation are still not clear and likely to be intricate. Studies revealed that transmural pressures or mechanical stretches could be sensed by the membrane sensor, such as integrins, ion channels, cytoskeleton and G‐proteins, and subsequently activated several intracellular signalling, including Ca^2+^ signalling, miRNAs, RhoA/ROCK, MAPK/ERK, PI3K/Akt, ROS formation, calcineurin/NFAT, actin polymerization, to modulate VSMC dedifferentiation and proliferation.^17^, ^25^, ^26^ Our previous study demonstrated that ion‐channel remodelling and elevated intracellular Ca^2+^ in cerebral VSMCs play a role in microgravity‐induced cerebrovascular adaptation.^6^, ^15^, ^19^, ^27^ Studies revealed that both L‐type VDCC and T‐type VDCC channels could be directly activated by increased blood pressure or mechanical stretch and lead to the Ca^2+^ influx into VSMCs.^18^, ^28^, ^29^ Interestingly, studies showed that contractile VSMCs are characterized by low resting cytosolic Ca^2+^ and a rapid transient change in intracellular Ca^2+^ concentration, whereas synthetic VSMCs are characterized by the sustained elevation of basal cytosolic Ca^2+^ and a long‐lasting intracellular Ca^2+^ oscillation.^12^, ^30^ Recent studies demonstrated that T‐type VDCCs played an important role in the sustained Ca^2+^ entry into VSMCs, which suggested that T‐type VDCCs may be responsible for VSMC dedifferentiation. In addition, it has been hypothesized that low arterial pressures (ranging from 40‐80 mm Hg) could activate T‐type VDCCs and then maintain the myogenic tone possibly via a low window‐type Ca^2+^ current, at which the VSMC membrane potentials are relatively hyperpolarized and L‐type VDCCs are inactive.^25^, ^28^ Therefore, T‐type VDCCs are more sensitive to low arterial pressure and are considered as an early‐response element to the increased blood pressure or mechanical stretch. Besides, T‐type VDCCs has been demonstrated to be associated with G1 and S phases of cell cycle, which is the necessary signalling for cell growth, proliferation, division and vascular neointima formation.^31^, ^32^ For example, it has been reported that T‐type Ca_V_3.1 channel promotes the IGF‐I induced‐cell cycle in pulmonary arterial VSMCs through the Ca^2+^‐dependent mitogenic signalling cascades.^32^ Both Ca_V_3.1 and Ca_V_3.2 channels are responsible for T‐type VDCC currents in VSMC.^33^ The present work showed that T‐type VDCCs currents in cerebral VSMCs of simulated microgravity rats were significantly increased, as a result of increasement of Ca_V_3.1, not Ca_V_3.2, channels (Figure 4). Similarly, the expressions of T‐type Ca_V_3.1 continuously increased during the process of VSMC dedifferentiation in vitro (Figure 5A‐C). Furthermore, we found that inhibition of T‐type Ca_V_3.1 channels by siRNA or antagonist (mibefradil) significantly suppressed the dedifferentiation and proliferation of cultured VSMCs (Figure 5D‐G). All these results from animal and cell studies clearly indicated that T‐type Ca_V_3.1 channel promoted the VSMC dedifferentiation and proliferation, which revealed one of the underlying mechanisms in simulated microgravity and general conditions.

Vascular smooth muscle cells phenotype, which modulated by Ca^2+^ signalling, is defined by Ca^2+^‐dependent transcription factors.^17^ Calcineurin (also called protein phosphatase 2B, PP2B) is a Ca^2+^/calmodulin‐activated serine/threonine phosphatase, which is responded to the sustained elevations of intracellular Ca^2+^. Activation of calcineurin could directly dephosphorylates NFAT transcription factors within the cytoplasm. Subsequently, the dephosphorylated NFAT would translocate to the nucleus and then mediate calcium‐inducible gene expression patterns.^12^, ^30^ Therefore, calcineurin participates in the transduction of extracellular signals to the nucleus by targeting NFAT transcription factors. The calcineurin/NFAT pathway is considered to be one of the most important Ca^2+^‐dependent signalling in VSMC dedifferentiation.^12^, ^17^ The present work showed that simulated microgravity not only induced the activation of T‐type Ca_V_3.1 channel, but also promoted the downstream calcineurin activity (Figure 6A). Five members of NFAT family have been identified up to now, NFATc1 (NFATc or NFAT2), NFATc2 (NFATp or NFAT1), NFATc3 (NFAT4 or NFATx), NFATc4 (NFAT3) and NFAT5.^17^ We found that simulated microgravity only increased nuclear translocation of NFATc3 among NFAT members that expressed in VSMC (Figure 6B, 6). In addition, silencing or blocking of Ca_V_3.1 inhibited activity of calcineurin/NFATc3 pathway (Figure 6D‐G) and suppressed VSMC dedifferentiation and proliferation (Figure 5D‐G). These results suggested that Ca_V_3.1 was involved in the VSMC dedifferentiation and proliferation by modulating calcineurin/NFATc3 activation.

MiRNAs are small non‐coding RNAs that negatively regulate mRNA stability or protein translation by binding to the 3′UTR of the target mRNA, which leads to the degradation of target mRNA or inhibition of translation in most cases.^34^ A series of specific miRNAs, including miR‐21, miR‐26a, miR‐206, miR‐23b, miR‐153/223, miR‐221/222, miR‐144/451 and miR‐143/145, were proved to be highly expressed in VSMCs and regarded as the upstream signalling of VSMC dedifferentiation.^8^, ^35^, ^36^ For example, miR‐145 has been demonstrated to promote VSMC differentiated contractile phenotype by targeting multiple factors, such as myocardin, Kruppel‐like transcription factors 4 and 5, actin dynamics, Ca^2+^/calmodulin‐dependent protein kinase IIδ (CamKIIδ) and angiotensin converting enzyme.^8^, ^37^ It has also been reported that miR‐145 could control the expression of L‐type Ca_V_1.2 channel by targeting CamKIIδ and subsequently regulate the stretch‐induced dedifferentiation in smooth muscle cells.^18^, ^26^ The present study showed that T‐type Ca_V_3.1 channel was subjected to a post‐transcriptional regulation in rat cerebral VSMCs under simulated microgravity (Figure 4A‐C), suggesting a potential role for miRNAs in it. However, there is no evidence indicating any miRNA could target T‐type Ca_V_3.1 channel. The present work confirmed that the potential Ca_V_3.1 related miR‐137, which predicted by bioinformatics, was significantly reduced during the process of VSMC dedifferentiation in vivo and in vitro (Figure 7A‐D). Moreover, we identified that miR‐137 could directly bind to 3’UTR of T‐type Ca_V_3.1 and downregulate its expression by using dual‐luciferase report assay and the gain‐ and loss‐of‐function approaches (Figure 8A‐D). The studies about miR‐137 showed that it is related to the regulation of cell proliferation and differentiation in neural cells, embryonic stem cells and various human cancer cells.^38^, ^39^, ^40^ For example, miR‐137 has been reported to target cell division cycle 42 to decrease proliferation, invasion and G0/G1 cell cycle progression in colorectal cancer cells.^40^ However, little is known about the role of miR‐137 in cardiovascular disease. In the present work, we found that inhibition of miR‐137 markedly promoted NFATc3 nuclear translocation, cell dedifferentiation and proliferation of cultured VSMCs, whereas the opposite effect was obtained through the overexpression of miR‐137 (Figure 7F, H). Finally, we demonstrated that blockage of T‐type Ca_V_3.1 channels by mibefradil could alleviate the dedifferentiation and proliferation of VSMC induced by miR‐137 downregulation (Figure 8E‐G). These results validated that miR‐137/Ca_V_3.1 axis was involved in the VSMC dedifferentiation and proliferation.

**Figure 9:**
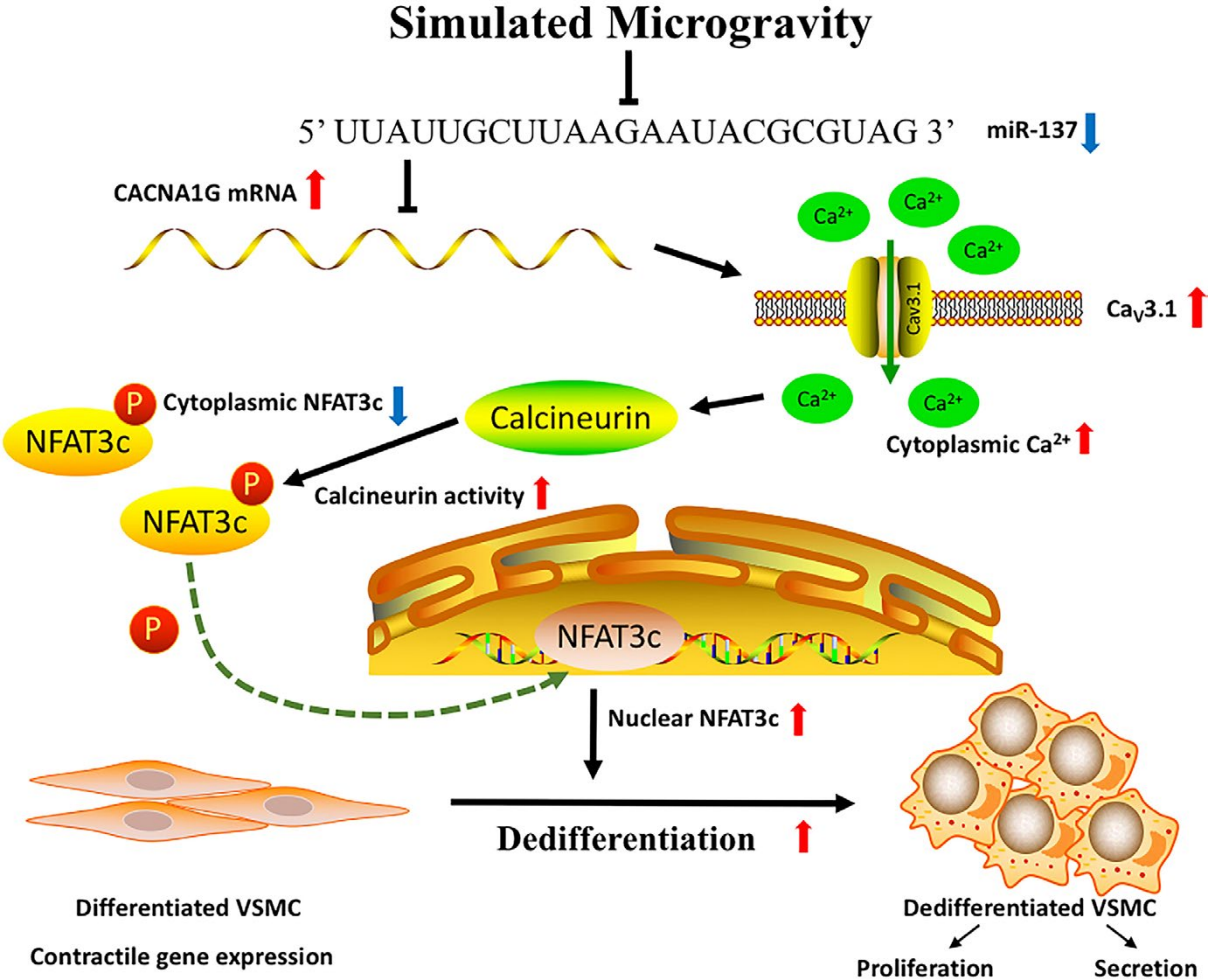
Schematic representation of the miR‐137‐Ca_V_3.1‐calcineurin‐NFATc3 signalling pathway in VSMC dedifferentiation and proliferation. In this mechanism, miR‐137 is downregulated in cerebral VSMCs under simulated microgravity conditions. As a direct target of miR‐137, Ca_V_3.1 is upregulated and the T‐type VDCC currents are enhanced. Due to Ca^2+^ entry through Ca_V_3.1 channel, the Ca^2+^ sensitive calcineurin activity is activated, more phosphorylated NFATc3 are dephosphorylated by calcineurin, and then translocate to nucleus to promote VSMC dedifferentiation and proliferation

As summarized in Figure 9, we demonstrated that miR‐137 and its target T‐type Ca_V_3.1 channel modulate dedifferentiation and proliferation of cerebral VSMCs in simulated microgravity rats by regulating calcineurin/NFATc3 pathway. This finding provides a novel mechanism of microgravity‐induced cerebrovascular adaptation and contributes to developing novel approaches as effective countermeasures against microgravity exposure. In particular, our study also has implications for VSMC dedifferentiation and proliferation in general conditions.

## CONFLICT OF INTEREST

The authors declare no competing financial interest.

## AUTHOR CONTRIBUTIONS

BZ, LC, HM and MX designed the experiments. BZ, LC and JS performed all experiments. YB and JM established animal model. JC analysed all data. BZ and MX drafted the manuscript.

## Data Availability

The data that support the findings of this study are available from the corresponding author upon reasonable request.
